# AddaVax-Adjuvanted H5N8 Inactivated Vaccine Induces Robust Humoral Immune Response against Different Clades of H5 Viruses

**DOI:** 10.3390/vaccines10101683

**Published:** 2022-10-09

**Authors:** Feixia Gao, Xueying Liu, Yudong Dang, Peng Duan, Wenting Xu, Xin Zhang, Shilei Wang, Jian Luo, Xiuling Li

**Affiliations:** Shanghai Institute of Biological Products, Shanghai 200052, China

**Keywords:** H5N8 vaccine, AddaVax, immunogenicity, hemagglutination inhibition, neuraminidase inhibition, microneutralization

## Abstract

Since some cases of human infections with H5N8 avian influenza virus have been reported and caused great concern in recent years, it is important to develop an effective vaccine for human use to prevent a potential H5N8 pandemic. In the present study, a vaccine candidate virus based on newly human-infected A/Astrakhan/3212/2020 H5N8 virus was constructed by reverse genetics (RG) technology. The immunogenicity of H5N8 whole virion inactivated vaccine was evaluated by various doses of vaccine antigen formulated with squalene-based adjuvant (AddaVax), aluminum hydroxide (Al(OH)_3_) or without adjuvant in mice. The results showed AddaVax-adjuvanted H5N8 inactivated vaccine could stimulate the mice to produce a stronger protective immune response with higher titers of IgG antibodies, hemagglutination inhibition (HI), neuraminidase inhibition (NI) and microneutralization (MN) antibodies than vaccine formulations with Al(OH)_3_ adjuvant or without adjuvant, and achieve a dose-sparing effect. Moreover, the AddaVax-adjuvanted formulation also exhibited potent cross-reactive response in HI antibodies against different clades of H5 viruses. A significant correlation and a curve fitting among HI, NI and MN were found by the correlation analysis to predict the protective effect of the vaccine. With these findings, our study demonstrates that AddaVax adjuvant can enhance the immunogenicity of H5N8 inactivated vaccine remarkably, and proposes an effective strategy for dealing with a potential H5N8 virus pandemic.

## 1. Introduction

Highly pathogenic avian influenza (HPAI) viruses of H5Nx originated from A/goose/Guangdong/1/1996-like avian influenza virus in 1996. The HPAI A/H5 avian influenza virus lineage has diversified into ten clades (0–9) and multiple subclades. In particular, clade 2.3.4.4 has further evolved into eight subclades (a–h) since 2019 [1,2]. H5Nx viruses have continuously and rapidly spread to many countries, resulting in a significant threat to human health and economic losses [3].

Most of the classified H5Nx clades and subclades can cause human infection, especially the H5N1 and H5N6 subtypes, which have been reported to infect humans [4]. Since 2020, the highly pathogenic H5N8 avian influenza virus has occurred in poultry in at least 46 regions [5]. Furthermore, in early December of 2020, Russia reported seven human cases of H5N8 infection for the first time. H5N8 virus infection has become a major problem in poultry breeding and avian wildlife safety, and poses a potential threat to global public health [5,6]. Therefore, it is urgent to prepare an effective H5N8 vaccine for the control of a potential pandemic outbreak.

The whole-virion inactivated influenza vaccine is the main strategy for an avian influenza vaccine design for human use, while the development of a vaccine candidate virus is essential for pandemic preparedness. It has been found that the HA of various avian influenza viruses contains multi-basic cleavage sites that are lethal to chickens, such as arginine (R) and lysine (K) sites in H5Nx and H7Nx [7], and REKRRKR/GLF in H5N8 [8]. After deleting the polybasic amino acids at the HA cleavage site of the HPAI parent virus, the new modified HA, along with the NA of the wild virus and the six internal segments of the backbone (such as A/PR/8/1934 H1N1 virus) is frequently used to generate a new low pathogenic virus as a vaccine candidate virus by reverse genetics (RG) [9,10].

During the preparation of a pre-pandemic influenza vaccine, improving the immunogenicity of the vaccine and saving the dose of antigens need to be considered. Adjuvants (such as Al(OH)_3_, AS03 and MF59, etc.) can improve, regulate and expand the immune response, thereby increasing the efficacy of vaccines [11,12,13]. Furthermore, adjuvants including oil-in-water emulsions have shown to induce higher level of immune response for influenza vaccines than traditional aluminum hydroxide adjuvants [14]. AddaVax is a squalene-based oil-in-water nanoemulsion based on the MF59 formulation, which is easier to mix with antigens than Freund’s adjuvant in order to prepare influenza vaccine formulations, and activates Th1/Th2 response to enhance immunity [15].

In this study, a vaccine candidate virus based on newly human-infected A/Astrakhan/3212/2020 H5N8 strain was constructed by RG. Meanwhile, we aimed to determine the immunogenicity of H5N8 inactivated vaccine formulated with different adjuvants, by measuring the HI, MN and NI antibodies and also its cross-reacting activity against different clades of H5 viruses. The results demonstrated that addition of the AddaVax adjuvant has a strong immunopotentiating- and therefore dose-sparing effect. 

## 2. Methods and Materials

### 2.1. Cell Lines and Strains

Human embryonic kidney 293T cell line (ATCC^®^ CRL-3216™) and canine kidney cells (Madin-Darby Canine Kidney, MDCK, ATCC^®^ CCL-34, USA) were maintained in DMEM medium (Gibco, ThermoFisher, Grand Island, NY, USA, Cat. No. 41500) containing 10% fetal bovine serum (Gibco, Cat. No. 25200) in incubator at 37 °C with 5% CO_2_.

Vaccine candidate virus A/Vietnam/1194/2004 NIBRG-14(H5N1) (clade 1, obtained from the UK National Institute of Biological Standard and Control (NIBSC, UK)) and A/Hubei/29578/2016(H5N6)-RG (clade 2.3.4.4d, obtained from the China National Influenza Center) are preserved by Shanghai Institute of Biological Products (Shanghai, China).

### 2.2. Plasmid Construction and Vaccine Candidate Virus Rescue

Six internal genes (PB1, PB2, NP, NA, M and NS) were derived from X-157 [16], a high-yielding candidate virus backbone. The HA (GenBank: UJS29065.1) and NA (GenBank: UJS29066.1) genes of A/Astrakhan/3212/2020 (H5N8) virus (clade 2.3.4.4b) were synthesized (SHENGGONG), and the polybasic cleavage sites in HA were modified (see Table 1). Eight influenza virus genes were cloned into the pHW2000 vector for rescuing the vaccine candidate H5N8 virus by reverse genetics. After a 48 h incubation, the supernatant was passaged to 10-day-old specific-pathogen-free eggs to amplify the generated virus [17,18]. 

### 2.3. Vaccine Preparation

Vaccine candidate viruses were inoculated into embryonated chicken eggs (10–11 days old) and incubated at 34 °C for 60 h. After incubation, the allantoic fluid was harvested and centrifugated at 1800× *g* for 30 min. Then, the virus solution was inactivated with 0.01% formaldehyde and concentrated five times via cross-flow ultrafiltration with a 500 kDa membrane cassette. The inactivated virus was further purified with Capto Core 700 resin (GE Healthcare) in AKTA Purifier 100 system. Finally, the purified bulk was filtered with a 0.22 μm Sartobran P filter and stored at 4 °C. 

A/Vietnam/1194/2004 NIBRG-14(H5N1) virus obtained from NIBSC was also inoculated into embryonated chicken eggs, and then the allantoic fluid was harvested and centrifugated. The following procedures of the H5N1 vaccine preparation were the same as the H5N8 vaccine.

For quantitation of haemagglutinin in the vaccine candidate virus, the virus concentrates of H5N8 and H5N1 viruses were deglycosylated using PNGase F and analyzed by SDS-PAGE. The amount of HA as a percentage of total protein was then calculated by dividing the ‘total HA’ by the ‘total protein’ [19]. The total proteins of the purified virus concentrates were assayed using a BCA protein assay.

### 2.4. Vaccine Immunization

The mice study protocol (NO. 2021005) was approved by the laboratory animal management committee and the laboratory animal ethics and welfare protection group of the Shanghai Institute of Biological Products.

Six-week-old BALB/c mice (*n* = 5 per group) were intramuscularly vaccinated twice at day 0 and day 21 with an H5N8 inactivated vaccine (50 μL/dose) at doses of 0.015 μg, 0.15 μg or 1.5 μg HA antigen containing 0.5 mg/mL of Al(OH)_3_ (Life science, Beijing, China) or 25 μL of AddaVax ™ (InvivoGen, San Diego, CA, USA) adjuvant or non-adjuvant; control mice were mock-vaccinated (PBS, 50 μL per dose). Al(OH)_3_ and AddaVax were used according to the manufacturer’s instructions (adjuvant: antigen = 1:1). In addition, BALB/c mice (*n* = 5 per group) were also intramuscularly vaccinated twice at day 0 and day 21 with 0.15 μg HA antigen of A/Vietnam/1194/2004 NIBRG-14(H5N1) inactivated vaccine alone (50 μL/dose) or in combination with 25 μL of Al(OH)_3_ adjuvant. Serum samples were collected on day 21 and day 42 and stored at −20 °C for serological analysis.

### 2.5. Enzyme-Linked Immunosorbent Assay (ELISA)

To determine total H5N8-specific IgG in immunized mouse sera, microtiter plates (Corning, NY, USA) were coated with 100 μL/well of H5N8 inactivated vaccine at 4 °C overnight, and blocked with 5% bovine serum albumin (BSA) in PBS for 1 h at 37 °C. Sera were tested at a starting dilution of 1:100 and applied to wells for 1 h at 37 °C, followed by incubation with rabbit anti-mouse IgG-peroxidase antibody for 1 h at 37 °C. The plates were developed using TMB, following the addition of 2 M H_2_SO_4_ to stop the reaction [20]. The means + 2 × SD of the naive group were used for the Ab-positive cut-off values set.

### 2.6. Hemagglutination Inhibition (HI) Assay

The functional antibodies that inhibit hemagglutination was assayed by the hemagglutination inhibition (HI) assay [21]. Serum was tested at 2-fold dilution with an initial dilution of 1:10. A titer of 5 was recorded if there was no inhibition at a serum dilution of 1:10.

### 2.7. Microneutralization Assay (MN) 

The detection of influenza virus neutralizing antibody titers was performed according to the protocol recommended by the World Health Organization. Briefly, each serum treated with receptor-destroying enzyme (RDE) was diluted 2-fold with DMEM including 5 μg/mL TPCK-treated trypsin (Sigma, MO, USA). An equal volume of virus at a concentration of 100 TCID_50_ was added and incubated at 37 °C for 1 h, then the mixture samples were added into 2 × 10^4^ MDCK cells each well and incubated for 72 h at 37 °C, 5% CO_2._ Freshly prepared 1% turkey erythrocytes were added, and were observed 1 h later. Neutralizing titers per milliliter of serum were calculated by the Reed and Muench method [22].

### 2.8. Neuraminidase Inhibition (NI) Assay

The NI assay was performed according to the protocol. The median inhibitory concentration (IC50) was calculated as the reverse dilution of the serum, resulting in a 50% inhibition of NA activity [13,23].

### 2.9. Statistics

The data were counted and analyzed on GraphPad Prism 8.0 software. T-test was used for comparison between the two groups, and one-way ANOVA was used for comparison between multiple groups; *p* < 0.05.

## 3. Results

### 3.1. Generation of Vaccine Candidate Virus and Preparation of H5N8 Inactivated Vaccine

**Figure 1:**
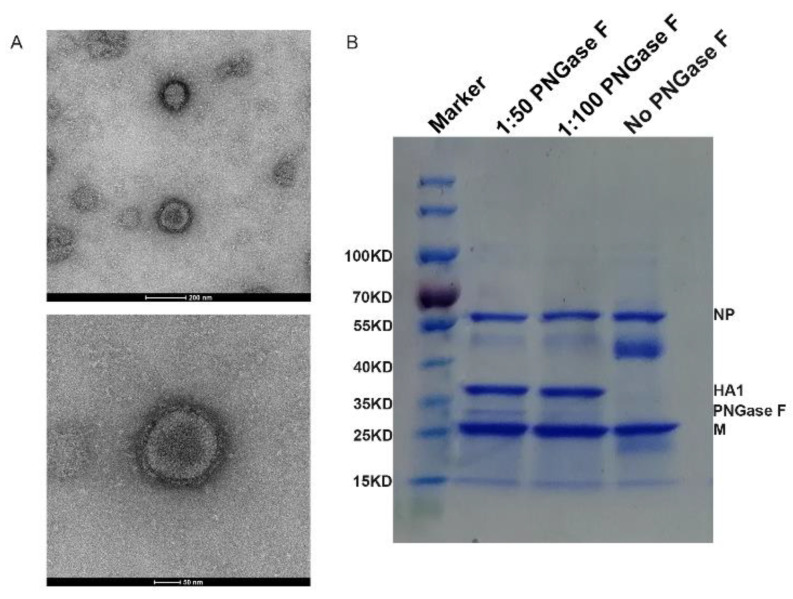
Characterization and identification of the inactivated whole-virus vaccine. (**A**) The electron micrograph of H5N8 inactivated whole viruses. (**B**) SDS-PAGE analysis of 3μg total protein in H5N8 virus vaccines with or without PNGase F treating (1:50 or 1:100 ratio). Purified HA protein of indicated amount was loaded to calibrate the HA content; the gel was stained with colloidal blue; images of SDS-PAGE were acquired by Bio-RAD software (ChemiDoc XRS+, 1708265). Each sample was tested in triplicate.

The HA and NA genes of the A/Astrakhan/3212/2020 H5N8 virus were synthesized. LREKRRKRGLF was modified to LRETRGLF at the HA cleavage site (Table 1) and cloned into the vector pHW2000. According to the method in the literature [18], RG (6 + 2) was used to combine the mutated HA gene, the NA wild-type gene and six internal genes of influenza virus to obtain a vaccine candidate virus. We found that the vaccine candidate virus could achieve high titer in chicken embryos, with a titer of 8.5 logTCID_50_/mL and an HA titer of 12 log_2_ HAU/50μL. The transmission electron microscope observation showed the H5N8 inactivated vaccine primary exhibited the small round (5–20 nm) structures. (Figure 1A). In order to evaluate the abundance of HA vaccine antigen, deglycosylation of the HA was achieved using PNGase F, and deglycosylated samples were analyzed by SDS–PAGE [19]. (Figure 1B). The statistical result showed that HA protein was estimated to constitute 30.1% of the total protein of the H5N8 inactivated vaccine.

### 3.2. Immune Response Induced by H5N8 Inactivated Vaccine in Mouse

To investigate the immunogenicity and optimization of vaccine formulation, a series of vaccinations were performed in mice to address the dose-response and adjuvant effects of H5N8 vaccine. Mice were immunized intramuscularly twice with various doses of H5N8 inactivated vaccine alone or in combination with Al(OH)_3_ or AddaVax adjuvants. The sera from the mice were collected at days 21 and 42 after immunization for further detection.

**Figure 2:**
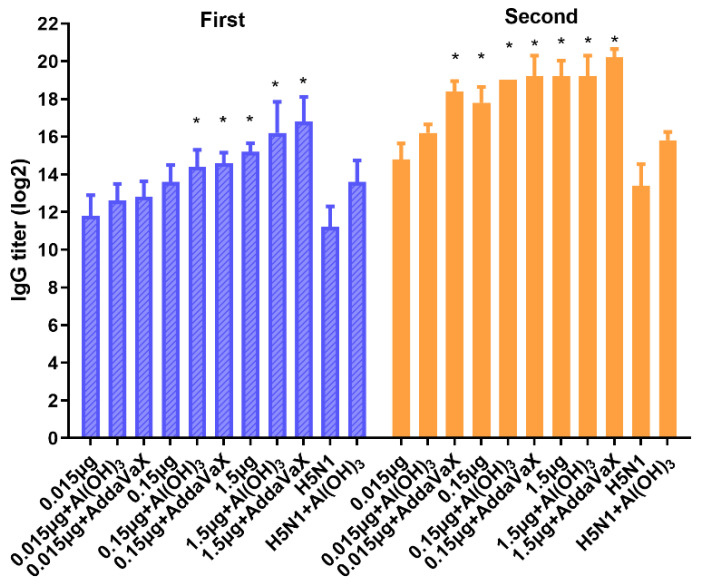
Humoral immune response elicited by H5N8 inactivated vaccine with or without adjuvants. BALB/c mice (*n* = 5) were immunized twice with various doses of H5N8 inactivated vaccines with or without adjuvants. The titers of virus specific IgG were detected by ELISA at day 21 post the first or last immunization. An amount of 2 μg/mL of H5N8 inactivated vaccine was used for plate coating. Comparing the vaccination effect among various formulations, an asterisk * indicates a significant difference (*p* < 0.05) detected by one-way ANOVA.

The specific IgG antibodies in the mice were evaluated firstly after vaccination. As expected, the antibody responses induced by the H5N8 vaccine groups after the second vaccination were stronger than the first vaccination. Moreover, the AddaVax-adjuvanted H5N8 vaccines produced higher IgG antibodies than those produced by the Al(OH)_3_-adjuvanted or unadjuvanted H5N8 vaccines (*p* < 0.05) (Figure 2), which suggested that the AddaVax-adjuvanted H5N8 vaccines might be a superior formulation to elicit a significant humoral immune response specific to the H5N8 virus.

**Figure 3:**
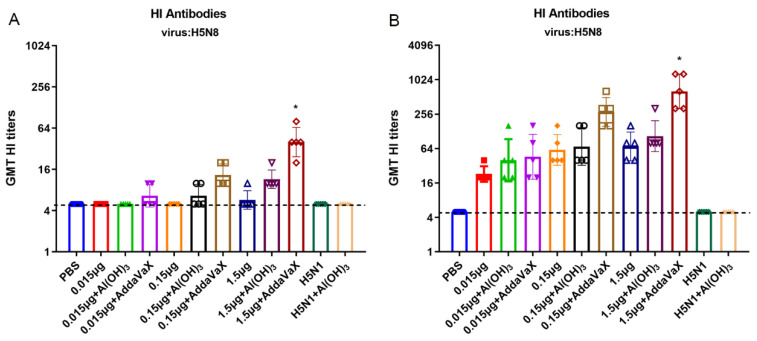
HI titers elicited by H5N8 inactivated vaccine with or without adjuvants. BALB/c mice (*n* = 5) were immunized twice with various doses of H5N8 inactivated vaccines with or without adjuvants, or with PBS as a control. (**A**) Serum HI antibodies against H5N8 virus at day 21 post the first immunization were determined by HI assay. (**B**) Serum HI antibodies against H5N8 virus at day 21 post the last immunization were determined by HI assay. Comparing the vaccination effect among various formulations, the asterisk * indicates a significant difference (*p* < 0.05) detected by one-way ANOVA.

HI antibody is the gold standard for evaluating the immunogenicity of influenza vaccines. In our experiment, only 1.5 μg AddaVax-adjuvanted H5N8 vaccine could elicit HI titers of up to 80 after the first vaccination (*p* < 0.05), and the HI titers of the other vaccine groups were all lower than or equal to 10 (Figure 3A). After the second immunization, whether low-dose or high-dose of the vaccine antigen, the resulting HI titers illustrated that the adjuvants enhanced the immunogenicity of the H5N8 inactivated vaccine (Figure 3B). In addition, 1.5 μg AddaVax-adjuvanted H5N8 vaccine elicited the highest geometric mean titers (GMT) with HI titers of 1024 (*p* < 0.05), while the HI titers of the Al(OH)_3_-adjuvanted and unadjuvanted H5N8 vaccine were 256 and 128, respectively. More importantly, the group vaccinated with 0.15μg H5N8 vaccine combined with AddaVax adjuvant was found to have higher HI against H5N8 virus than the 1.5 μg unadjuvanted group. The data above indicate that the AddaVax adjuvant had immuno-enhancing effect in stimulating HI antibodies, as well as a better antigen-sparing effect. 

**Figure 4:**
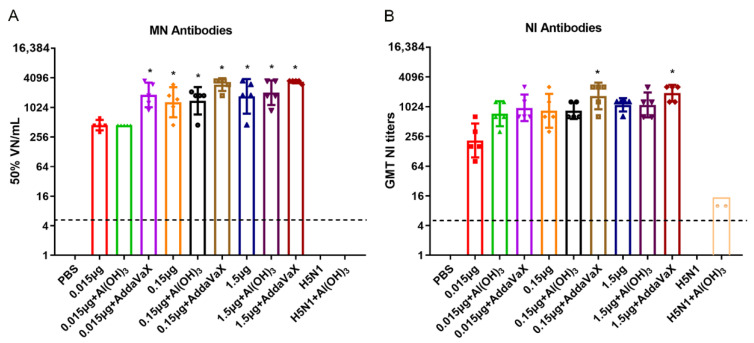
MN and NI titers elicited by H5N8 inactivated vaccine with or without adjuvants. BALB/c mice (*n* = 5) were immunized twice with various doses of H5N8 inactivated vaccines with or without adjuvants. (**A**) The MN titers against H5N8 virus in serum were analyzed by MN assay at day 21 post the last immunization. (**B**) The NI titers against H5N8 virus in serum were analyzed by NI assay at day 21 post the last immunization. The H5N8 whole-virus inactivated vaccine acted as the source of antigen. Comparing the vaccination effect among various formulations, an asterisk * indicates a significant difference (*p* < 0.05), by one-way ANOVA.

MN antibodies have been used to measure humoral immune responses to influenza, and more recently to antigenically characterize influenza viruses [24]. A microneutralization assay was performed to evaluate the virus-neutralizing ability of vaccine sera on MDCK cells. The results showed that H5N8 vaccine could induce MN antibodies against the homologous virus in a dose-dependent manner (*p* < 0.05). The MN antibodies induced by the AddaVax-adjuvanted H5N8 vaccine were higher than those induced by Al(OH)_3_ or non-adjuvant groups (Figure 4A).

Besides HI and MN antibodies, NI antibodies can provide cross-protection and may be a strong correlate of influenza-related disease severity [25]. For evaluating the NI antibodies induced by vaccine immunization, the ELLA assay was performed to measure the NI antibodies, which measured the amount of penultimate galactose that became available after the NA cleaved the sialic acid on the fetuin surface [26]. The results indicated that the H5N8 vaccines combined with AddaVax elicited significantly higher immunity compared to other groups, and NI antibody titers of 1.5 μg AddaVax-adjuvanted H5N8 vaccine were the highest, which reaching up to 1940 (GMT) (*p* < 0.05). The dose-dependent effects of vaccination on enhancing NI titers were not observed in the groups vaccinated with Al(OH)_3_-adjuvanted vaccine doses reaching 0.15 μg and 1.5 μg (Figure 4B).

As expected, all the H5N1 vaccines showed very low or no titers in MN and NI against the H5N8 virus, which suggesting that the H5N1 vaccine might not produce cross-neutralizing antibodies against the H5N8 virus (Figure 4).

In summary, these results suggested that the AddaVax-based adjuvant had great potential in improving the immunogenicity of H5N8 inactivated vaccine, being able to activate humoral immune response with higher levels of IgG, HI, NI and MN antibodies.

### 3.3. Cross-Reactive Antibody Response against Different Clades of H5 Viruses 

**Figure 5:**
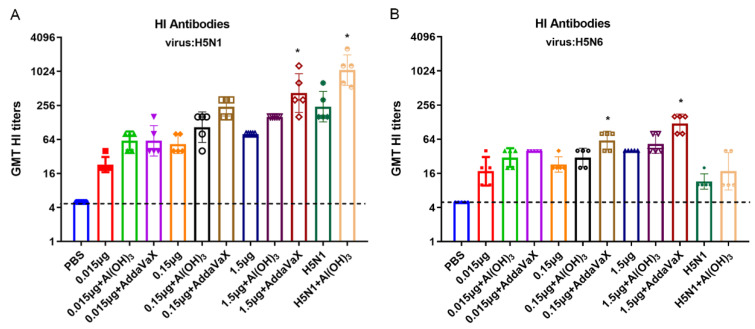
Cross-reactive neutralizing antibody against different clades of H5 viruses. Cross-reactive neutralizing antibody responses were determined by HI assay. (**A**) Antisera were analyzed for HI titers against H5N1 virus. (**B**) Antisera were analyzed for HI titers against H5N6 virus. Comparing the vaccination effect among various formulations, an asterisk * indicates a significant difference (*p* < 0.05) detected by one-way ANOVA.

The H5N6 and H5N8 viruses in H5Nx belong to the H5 clades 2.3.4.4d and 2.3.4.4b, and the HA homology between them reaches 93%. The H5N1 virus belongs to the H5 clade 1, and its homology with the HA of H5N8 reaches 90%. To investigate potential cross-protection of H5N8 vaccine against different clades of H5 viruses, HI titers were determined, and we found that the presence of AddaVax adjuvant substantially enhanced the H5N8 vaccine efficacy and elicited an adequate immune response against both H5-clade viruses. Nevertheless, the HI antibody titer induced by Al(OH)_3_-adjuvanted H5N8 vaccine against H5N1 virus was up to 160, while that induced against H5N6 virus was lower. The unadjuvanted H5N8 vaccine only detected a small number of HI antibody titers in the high-dose antigen group (Figure 5).

Additionally, H5N1 vaccines could elicit high HI antibody titers against homologous H5N1 virus, while vaccines combined with Al(OH)_3_ only induced a small amount of HI antibody titers against H5N6 virus, meanwhile failing to induce a cross reaction to H5N8 virus, as evidenced by the absence of HI titers (Figure 3 and Figure 5).

Overall, AddaVax-based adjuvant H5N8 vaccines induced not only antibody responses against homologous viruses, but also cross-reactions against different clades of H5 viruses, and even achieved antigen-sparing effects.

### 3.4. Correlation Analysis between Functional Antibodies

**Figure 6:**
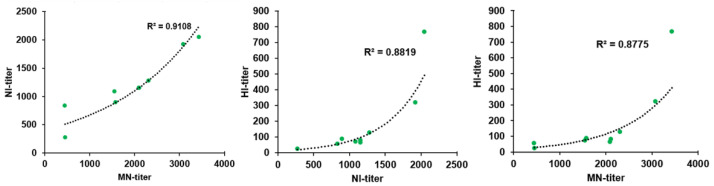
Correlation analysis among different antibody titers at day 21 post the last immunization. Correlation among HI/NI/MN antibody titers using the fitted curves. R^2^ indicates the goodness of the fit.

The three humoral immune parameters of HI, NI and MN antibodies can be used to evaluate the immunogenicity and immune efficacy of vaccines. In order to study their correlation, the data were subjected to curve fitting test, and there was a strong correlation among HI, NI and MN (Figure 6). The curves between NI and MN were fitting, showing an initial linear relationship, and implying equal sensitivity. The NI titers increased with the growth of MN titers, and vice versa. Additionally, there was also a good correlation among HI, NI and MN (R^2^ > 0.8), indicating that the three can be used as evaluation indices of immunogenicity. The increase in one antibody level was also related to corresponding increases in other antibody levels, which can be used as cross-reference indices for the evaluation of immunogenicity.

## 4. Discussion

The prevalence of the H5N8 virus has raised great concern since seven cases of human infection with H5N8 influenza viruses were reported in the Russian Federation in 2020. The most cost-effective way to prevent the spread of highly pathogenic avian influenza disease is to immunize humans through widespread vaccination. In this study, the HA gene of H5N8 virus was modified at the cleavage site, which can reduce the pathogenicity of avian influenza virus to avian embryos and poultry, and the vaccine candidate virus based on A/Astrakhan/3212/2020 H5N8 was constructed by RG. We examined an H5N8 inactivated vaccine, both with and without AddaVax adjuvant, at different dose levels, and found that the AddaVax adjuvant significantly enhanced HI, NI and MN antibody responses. Moreover, the AddaVax-adjuvanted H5N8 inactivated vaccine also elicited a certain degree of cross-reacting antibodies against different clades of H5 viruses. Our results illustrated that squalene-based adjuvant may confer a superior formulation for enhancing H5N8 vaccine efficacy, affording a dose-sparing effect.

Most clinical studies have shown that avian influenza vaccines are less immunogenic than seasonal influenza vaccines, or induce weaker immune memory in humans, thus requiring adjuvantation or double-dose administration to improve vaccine efficacy [27,28]. To our knowledge, Al(OH)_3_ adjuvant has resulted in an improvement of the immune response in humans [29]. In addition to Al(OH)_3_ adjuvant, the safety and efficacy of squalene-based immunogenic adjuvants such as MF59 have been well proved in many human clinical trials [30]. So far, it has been widely used to prepare the current licensed influenza virus vaccines. AddaVax is a squalene-based oil-in-water emulsion, similar to the MF59 adjuvant, that attracts immune cells to the injection site by stimulating local cytokine and chemokine production, and increases antigen trafficking and presentation, thus inducing significant neutralizing antibody responses to provide protection against viral infection in vaccinated animals [30,31,32]. Studies have shown that inactivated H3N2 vaccine with AddaVax and polyinosine: polycytidylic acid (PolyI:C) adjuvant elicit higher HI and IgG titers, producing a strong adaptive immune response and protection [33]. Furthermore, AddaVax stimulated higher antibody responses in mice than poly(l:C) and Al(OH)_3_ [34]. Similarly to previous studies, in our experiments, the H5N8 inactivated vaccines with different components were found to induce humoral immune responses in mice with higher titers of IgG, HI, NI and MN antibodies, of which the AddaVax-adjuvanted H5N8 inactivated vaccine elicited the highest antibody titers; thus, AddaVax is an effective immunomodulator for optimizing the formulation of the vaccine.

HI, NI and MN antibodies play an indispensable role in preventing viral infections [35]. The H5N8 virus in the present study was designated as clade 2.3.4.4b. The AddaVax-adjuvanted H5N8 inactivated vaccine induced higher HI antibody responses against homologous H5N8 virus or different clades of H5 viruses (H5N1 clade 1 and H5N6 clade 2.3.4.4d), indicating that the AddaVax-adjuvanted vaccines could provide cross-protection. This is consistent with previous studies, which also showed that the MF59-based vaccine could induce cross-neutralizing antibodies to confer protection against homologous and heterosubtype H5 strains [36,37], while Al(OH)_3_-adjuvanted H5N1 inactivated vaccine failed even to elicit NI and MN antibodies against H5N8 virus, suggesting that the H5N1 vaccine might not produce cross-neutralizing antibodies against the H5N8 virus.

Unexpectedly, the homologous HI antibodies stimulated by AddaVax-adjuvanted H5N8 vaccine were slightly lower than those stimulated by Al(OH)_3_-adjuvanted H5N1 inactivated vaccine. Since phenotypic diversity is one of the characteristics of H5 clade 2.3.4.4 viruses, genetic changes among different strains can induce antigenic alteration, and the antigenicity of viruses in different subclades varies widely, resulting in different HI activities among viruses [38]. Previous studies also demonstrated that the H5N8 vaccine could elicit robust seroprotective HI titers with multiple doses and adjuvants, in terms of the low immunogenicity of H5 HA compared with H1 HA [39]. Furthermore, in view of the accurate quantification of HA content in influenza vaccines, the difficulty with antigen payload was evident when HI titers were compared between the different H5N8 and H5N1 antigens. AddaVax-adjuvanted H5N8 inactivated vaccine elicited much higher titers of MN and NI antibodies against H5N8 virus than H5N8 vaccine with Al(OH)_3_ adjuvant or without adjuvant. Similarly, other studies have also illustrated that MN titers are much higher than HI titers in the clinical results of H5N1 vaccine combined with MF59 [37], and in seasonal influenza vaccines [24], which suggests that MN antibodies might be a more sensitive indicator of antibody-mediated protection.

Moreover, NA is more conservative among circulating strains. NI corresponds with cross-protection and is highly correlative with protective immunity [40,41]. Clinical studies showed that NI titer was negatively correlated with all four indicators of disease severity. It is proposed that NI should be used as another indicator to evaluate the protective effect of influenza vaccine in addition to HI antibody titer [25]. Therefore, even though the homologous HI titers induced by the AddaVax-adjuvanted H5N8 vaccine are slightly lower in this study, the NI titers are high enough to induce robust protective immunity, and may play an important role in inducing cross-reactive antibody response against different clades of H5 viruses. Recent studies have also displayed a strong correlation between functional antibodies and vaccine efficacy parameters. NI and MN data are more predictive in the lower range compared to HI data, and animals are not protected when NI and MN titers are undetectable [13]. According to correlation analysis on the HI, NI and MN data in this study, we found that these three functional antibodies have strong correlation and curve fitting, and HI, NI and MN can all be used as immune indicators for mutual reference evaluation. The higher serological neutralizing antibody titers of HI, NI and MN reveal that the H5N8 vaccine might have a broader efficacy and protect animals from infection. Nevertheless, the cellular immune response of the AddaVax-adjuvanted H5N8 inactivated vaccine in mice should be directly defined to further demonstrate the AddaVax adjuvant effects. Besides, a challenge study remains necessary to understand the efficacy of H5N8 vaccine more accurately.

More importantly, our observations that the HI antibody titers induced by the 0.15 μg AddaVax-adjuvanted H5N8 vaccine were much higher than those of the 1.5 μg antigen vaccine alone, which suggested that the squalene-based adjuvant economized on the use of the immunogenic antigen of H5N8 vaccine at least 10-fold. Previous studies have also indicated that AddaVax formula, which provided sufficient seroprotective titers, could achieve better antigen-sparing effect and cross-reactivity to H7 subtype virus [21]. Therefore, the squalene-based adjuvantation is a promising way to prepare for an effective H5N8 vaccine for a surge in demand.

## 5. Conclusions

In conclusion, this study conducted a preliminary evaluation of the immunogenicity and efficacy of the H5N8 inactivated vaccine, explored an available adjuvant, and evaluated the cross-reactivity. The data reported here show that the vaccine can induce the humoral immune response with higher titers of HI, NI and MN antibodies, and can provide a potent cross-reaction with different clades of H5 viruses, which may serve as a reference in preparing for a potential H5N8 pandemic. 

## Figures and Tables

**Table 1:** Hemagglutinin cleavage site of reassortant viruses.

A/Astrakhan/3212/2020 H5N8	Modification of Hemagglutinin Cleavage Site
Wild virus HA	L R E K R R K R ↓ G L F
	CTA AGA GAA AAG AGA AGA AAA AGA GGC CTG TTT
Modified HA	CTA AGA GAA ACG AGA GGC CTG TTT
	L R E T R G L F

↓: proteolytic cleavage site.

## References

[1] Huang C.-W., Chen L.-H., Lee D.-H., Liu Y.-P., Li W.-C., Lee M.-S., Chen Y.-P., Lee F., Chiou C.-J., Lin Y.-J. (2021). Evolutionary history of H5 highly pathogenic avian influenza viruses (clade 2.3.4.4c) circulating in Taiwan during 2015–2018. Infect. Genet. Evol..

[2] Evolution Working Group (2008). Toward a unified nomenclature system for highly pathogenic avian influenza virus (H5N1). Emerg. Infect. Dis..

[3] Nunez I., Ross T.M. (2019). A review of H5Nx avian influenza viruses. Ther. Adv. Vaccines Immunother..

[4] WHO (2022). Cumulative Number of Confirmed Human Cases for Avian Influenza A(H5N1) Reported to WHO, 2003–2021.

[5] Shi W., Gao G.F. (2021). Emerging H5N8 avian influenza viruses. Science.

[6] Jeong J.H., Kim E.-H., Lloren K.K.S., Kwon J.J., Kwon H.-I., Ahn S.J., Kim Y.-I., Choi W.-S., Si Y.-J., Lee O.-J. (2018). Preclinical evaluation of the efficacy of an H5N8 vaccine candidate (IDCDC-RG43A) in mouse and ferret models for pandemic preparedness. Vaccine.

[7] Jang Y., Seo S.H. (2022). H5 cleavage-site peptide vaccine protects chickens from lethal infection by highly pathogenic H5 avian influenza viruses. Arch. Virol..

[8] Liu Y., Liu C., Dang A., Sun S., Zhang D., Wang M., Chen F., Li Y., Xue R., Chen J. (2021). Pathological analysis and genetic characterization of the first outbreak H5N8 subtype avian influenza virus isolated from wild swan in Shandong, China. Transbound. Emerg. Dis..

[9] Mostafa A., Abdelwhab E.M., Mettenleiter T.C., Pleschka S. (2018). Zoonotic Potential of Influenza A Viruses: A Comprehensive Overview. Viruses.

[10] Moatasim Y., Kandeil A., Mostafa A., Kutkat O., Sayes M., El Taweel A., AlKhazindar M., AbdElSalam E., El-Shesheny R., Kayali G. (2021). Impact of Individual Viral Gene Segments from Influenza A/H5N8 Virus on the Protective Efficacy of Inactivated Subtype-Specific Influenza Vaccine. Pathogens.

[11] Reed S.G., Orr M.T., Fox C.B. (2013). Key roles of adjuvants in modern vaccines. Nat. Med..

[12] Giuseppe D.G., Rino R., Didierlaurent A.M. (2018). Correlates of adjuvanticity: A review on adjuvants in licensed vaccines. Semin. Immunol..

[13] De Jonge J., van Dijken H., de Heij F., Spijkers S., Mouthaan J., de Jong R., Roholl P., Adami E.A., Akamatsu M.A., Ho P.L. (2020). H7N9 influenza split vaccine with SWE oil-in-water adjuvant greatly enhances cross-reactive humoral immunity and protection against severe pneumonia in ferrets. npj Vaccines.

[14] Khurana S., Chearwae W., Castellino F., Manischewitz J., King L.R., Honorkiewicz A., Rock M.T., Edwards K.M., Del Giudice G., Rappuoli R. (2010). Vaccines with MF59 Adjuvant Expand the Antibody Repertoire to Target Protective Sites of Pandemic Avian H5N1 Influenza Virus. Sci. Transl. Med..

[15] Hawksworth D. (2018). Advancing Freund’s and AddaVax Adjuvant Regimens Using CpG Oligodeoxynucleotides. Monoclon. Antib. Immunodiagn. Immunother..

[16] Ramanunninair M., Le J., Onodera S., Fulvini A.A., Pokorny B.A., Silverman J., Devis R., Arroyo J.M., He Y., Boyne A. (2013). Molecular Signature of High Yield (Growth) Influenza A Virus Reassortants Prepared as Candidate Vaccine Seeds. PLoS ONE.

[17] Hoffmann E., Neumann G., Kawaoka Y., Hobom G., Webster R.G. (2000). A DNA transfection system for generation of influenza A virus from eight plasmids. Proc. Natl. Acad. Sci. USA.

[18] Liu L.Q., Li Z., Jiao M., Lu J., Zhou J.F., Li X.Y., Liu J., Guo J.F., Xiao N., Zhao X. (2020). Development and Assessment of Two Highly Pathogenic Avian Influenza (HPAI) H5N6 Candidate Vaccine Viruses for Pandemic Preparedness. Biomed. Environ. Sci..

[19] Shao M., Li J., Song Y.L., Cui X.Y., Yuan L.Y., Fang H.H. (2010). Development and Validation of Alternative Method for Determination of Haemagglutinin Content in Influenza Vaccine. Chin. J. Biol..

[20] Gao F., Yang T., Liu X., Xiong F., Luo J., Yi Y., Fan J., Chen Z., Tan W.-S. (2020). MiRNA Targeted NP Genome of Live Attenuated Influenza Vaccines Provide Cross-Protection against a Lethal Influenza Virus Infection. Vaccines.

[21] Tzeng T.-T., Chen P.-L., Weng T.-C., Tsai S.-Y., Lai C.-C., Chou H.-I., Chen P.-W., Lu C.-C., Liu M.-T., Sung W.-C. (2020). Development of high-growth influenza H7N9 prepandemic candidate vaccine viruses in suspension MDCK cells. J. Biomed. Sci..

[22] Reed L.J., Muench H. (1938). A simple method of estimating fifty per cent endpoints. Am. J. Epidemiol..

[23] Xiong F.-F., Liu X.-Y., Gao F.-X., Luo J., Duan P., Tan W.-S., Chen Z. (2020). Protective efficacy of anti-neuraminidase monoclonal antibodies against H7N9 influenza virus infection. Emerg. Microbes Infect..

[24] Heeringa M.L.B.S. (2020). Comparability of Titers of Antibodies against Seasonal Influenza Virus Strains as Determined by Hemagglutination Inhibition and Microneutralization Assays. J. Clin. Microbiol..

[25] Memoli M.J., Shaw P.A., Han A., Czajkowski L., Reed S., Athota R., Bristol T., Fargis S., Risos K., Powers J.H. (2016). Evaluation of Antihemagglutinin and Antineuraminidase Antibodies as Correlates of Protection in an Influenza A/H1N1 Virus Healthy Human Challenge Model. mBio.

[26] Couzens L., Gao J., Westgeest K., Sandbulte M., Lugovtsev V., Fouchier R., Eichelberger M. (2014). An optimized enzyme-linked lectin assay to measure influenza A virus neuraminidase inhibition antibody titers in human sera. J. Virol. Methods.

[27] Belshe R.B., Frey S.E., Graham I., Mulligan M.J., Edupuganti S., Jackson L.A., Wald A., Poland G., Jacobson R., Keyserling H.L. (2011). Safety and Immunogenicity of Influenza A H5 Subunit Vaccines: Effect of Vaccine Schedule and Antigenic Variant. J. Infect. Dis..

[28] Czajka H., Unal S., Ulusoy S., Usluer G., Strus A., Sennaroglu E., Guzik J., Iskit A.T., Dargiewicz A., Musial D. (2012). A phase II, randomised clinical trial to demonstrate the non-inferiority of low-dose MF59Â^®^-adjuvanted pre-pandemic A/H5N1 influenza vaccine in adult and elderly subjects. J. Prev. Med. Hyg..

[29] He P., Zou Y., Hu Z. (2015). Advances in aluminum hydroxide-based adjuvant research and its mechanism. Hum. Vaccines Immunother..

[30] O’Hagan D.T. (2007). MF59 is a safe and potent vaccine adjuvant that enhances protection against influenza virus infection. Expert Rev. Vaccines.

[31] O’Hagan D., Ott G., De Gregorio E., Seubert A. (2012). The mechanism of action of MF59—An innately attractive adjuvant formulation. Vaccine.

[32] Nyon M.P., Du L., Tseng C.-T.K., Seid C.A., Pollet J., Naceanceno K.S., Agrawal A., Algaissi A., Peng B.-H., Tai W. (2018). Engineering a stable CHO cell line for the expression of a MERS-coronavirus vaccine antigen. Vaccine.

[33] Nian X., Zhang J., Deng T., Liu J., Gong Z., Lv C., Yao L., Li J., Huang S., Yang X. (2021). AddaVax Formulated with PolyI:C as a Potential Adjuvant of MDCK-based Influenza Vaccine Enhances Local, Cellular, and Antibody Protective Immune Response in Mice. AAPS PharmSciTech.

[34] Luo K., Gordy J.T., Zavala F., Markham R.B. (2021). A chemokine-fusion vaccine targeting immature dendritic cells elicits elevated antibody responses to malaria sporozoites in infant macaques. Sci. Rep..

[35] Trombetta C.M., Montomoli E. (2016). Influenza immunology evaluation and correlates of protection: A focus on vaccines. Expert Rev. Vaccines.

[36] Versage E., van Twuijver E., Jansen W., Theeuwes A., Sawlwin D., Hohenboken M. (2021). Analyses of Safety Profile and Homologous Antibody Responses to a Mammalian Cell-Based, MF59-Adjuvanted, A/H5N1, Pandemic Influenza Vaccine across Four Phase II/III Clinical Trials in Healthy Children, Adults, and Older Adults. Vaccines.

[37] Chanthavanich P., Versage E., Van Twuijver E., Hohenboken M. (2021). Antibody responses against heterologous A/H5N1 strains for an MF59-adjuvanted cell culture–derived A/H5N1 (aH5N1c) influenza vaccine in healthy pediatric subjects. Vaccine.

[38] Antigua K.J.C., Choi W.-S., Baek Y.H., Song M.-S. (2019). The Emergence and Decennary Distribution of Clade 2.3.4.4 HPAI H5Nx. Microorganisms.

[39] Lee M.-S., Jang E.Y., Cho J., Kim K., Lee C.H., Yi H. (2019). Development and comparison of two H5N8 influenza A vaccine candidate strains. Arch. Virol..

[40] Rockman S., Brown L.E., Barr I.G., Gilbertson B., Lowther S., Kachurin A., Kachurina O., Klippel J., Bodle J., Pearse M. (2013). Neuraminidase-Inhibiting Antibody Is a Correlate of Cross-Protection against Lethal H5N1 Influenza Virus in Ferrets Immunized with Seasonal Influenza Vaccine. J. Virol..

[41] Zhou F., Hansen L., Pedersen G., Grødeland G., Cox R. (2021). Matrix M Adjuvanted H5N1 Vaccine Elicits Broadly Neutralizing Antibodies and Neuraminidase Inhibiting Antibodies in Humans That Correlate with In Vivo Protection. Front. Immunol..

